# Case report: Short-term eculizumab use in atypical HUS associated with Lemierre's syndrome and post-infectious glomerulonephritis

**DOI:** 10.3389/fmed.2023.1167806

**Published:** 2023-05-03

**Authors:** Sanober Sadiq, Anatoly Urisman, Onur Cil

**Affiliations:** ^1^Division of Nephrology, Department of Pediatrics, University of California, San Francisco, San Francisco, CA, United States; ^2^Department of Pathology, University of California, San Francisco, San Francisco, CA, United States

**Keywords:** eculizumab discontinuation, *Fusobacterium*, *Eikenella*, prognosis, atypical HUS

## Abstract

Atypical hemolytic uremic syndrome (aHUS) is a rare disease caused by genetic abnormalities, infections, autoimmune diseases, drugs, and malignancies. Anti-C5 monoclonal antibody eculizumab is the mainstay of treatment of aHUS caused by the genetic defects of the alternative complement pathway. However, the utility of eculizumab in non-genetic forms of aHUS and the timing of treatment discontinuation remain controversial. Here, we report successful short-term eculizumab use in two young adult patients with aHUS due to rare infectious and autoimmune etiologies: Lemierre's syndrome and post-infectious glomerulonephritis, respectively. Eculizumab was rapidly discontinued in both patients with no aHUS recurrence during long-term follow-up. Considering its favorable safety profile with appropriate meningococcal prophylaxis, eculizumab can be considered as a treatment option for non-genetic aHUS.

## Introduction

Hemolytic uremic syndrome (HUS) is characterized by microangiopathic hemolytic anemia, thrombocytopenia, and kidney failure. Most cases of childhood HUS are due to diarrheal disease caused by Shiga toxin-producing *E. coli* (STEC) infections. *S. pneumonia* and H1N1/Influenza A are also well-known infectious causes of HUS. Atypical HUS (aHUS) is a term used to describe other forms of HUS which are classified into the following categories: complement-mediated, non-complement-mediated, metabolism-associated, coagulation-mediated, secondary, transplant-associated, drug-induced, and pregnancy-induced (1). Eculizumab is an anti-C5 monoclonal antibody that blocks the activation of the terminal complement pathway and is currently the main treatment for aHUS caused by the genetic defects of the alternative complement pathway (2). Although some anecdotal studies report the efficacy of eculizumab in STEC-HUS and non-genetic aHUS (3, 4), its use in these conditions and the timing of treatment discontinuation remain controversial (2). Here, we report the efficacy and successful rapid discontinuation of eculizumab in two young adult patients with severe aHUS due to rare infectious and autoimmune etiologies.

### Patient 1

**Figure 1:**
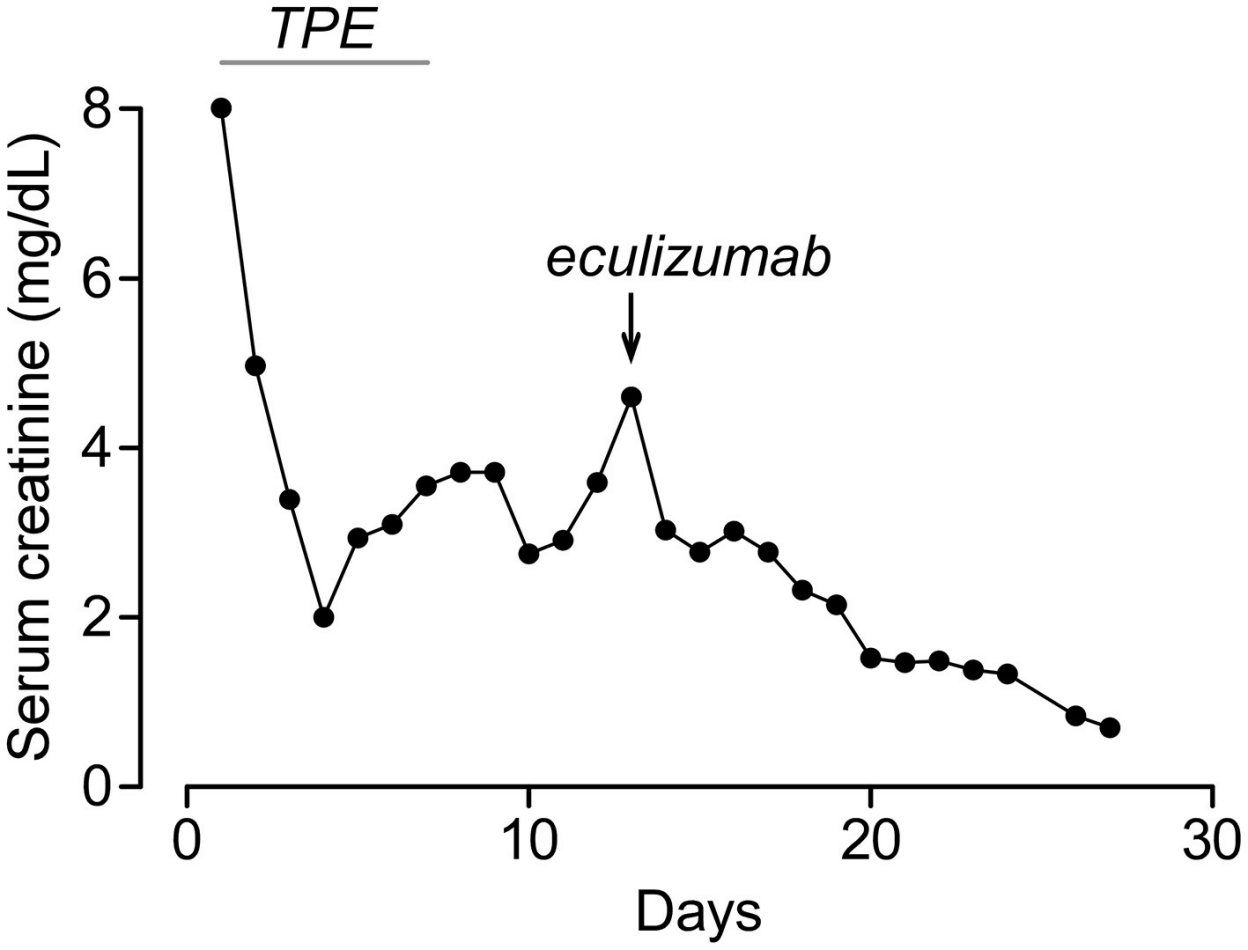
Serum creatinine of patient 1 during hospitalization. Timing of plasma exchange (TPE) and eculizumab treatments are indicated.

A 22-year-old African-American male patient presented with a sore throat, headaches, and back pain. A physical examination showed an ill-appearing young male patient with altered mental status. Vital signs were significant for normal temperature, tachycardia (122 bpm), tachypnea (26 per min), and mild hypertension (133/63 mmHg). Laboratories were notable for elevated BUN/creatinine (136/9.5 mg/dL), thrombocytopenia (12 × 10^9^/L), anemia (11.8 g/dL), elevated LDH (1,153 U/L), low haptoglobin (<6 mg/dl), and schistocytes in peripheral smear. Initially, he was thought to have thrombotic thrombocytopenic purpura (TTP) due to the presence of neurological signs and was started on therapeutic plasma exchange (TPE). During the first session, he became febrile and hypotensive. He had ongoing oliguria despite IV fluids and vasopressors and was started on continuous renal replacement therapy (CRRT). His autoimmune workup was unremarkable including normal C3, C4, and CH50 and negative ANCA, anti-dsDNA, anti-GBM, and anti-SCL70. He developed left arm swelling on Day 2 of admission and had a neck ultrasound that showed a non-occlusive thrombus in the left internal jugular vein. Around the same time, his peripheral blood culture came back positive for *Fusobacterium necrophorum* and *Eikenella corrodens*. Based on the ultrasound and culture results, he was diagnosed with Lemierre's syndrome (septic thrombophlebitis of internal jugular vein) and was started on antibiotics. Although he was initially thought to have TTP, his ADAMTS13 activity was found normal at 90%, which suggested that his thrombotic microangiopathy (TMA) was caused by aHUS. His TMA workup included normal Factor I, Factor H, and Factor B levels, normal leukocyte MCP expression, and negative anti-CFH antibodies. The patient clinically improved with antibiotics and TPE. After becoming hemodynamically stable, his CRRT was stopped on Day 4 and his platelets normalized on Day 7. However, he continued to have marked renal dysfunction and oliguria non-responsive to diuretics and required intermittent hemodialysis. His aHUS genetic testing (*C3, CFB, CFH, CFI, MCP, CFHR1, CFHR3, CFHR5, DGKE*, and *THBD*) showed homozygous 84-kb deletion in *CFHR1-CFHR3*. This deletion is typically associated with anti-CFH antibodies (5); however, his antibodies were negative on two occasions including the admission day. Although his platelets normalized, he had persistent anemia (Hb: 7.6 g/dL) with elevated LDH (247 U/L, normal <200) and worsening creatinine (2.9 on Day 5 to 4.6 mg/dL on Day 13), and he was started on eculizumab treatment on Day 13. After eculizumab, there was a rapid improvement in his creatinine (Figure 1), and his hemodialysis treatment was stopped. His creatinine improved to 0.8 mg/dL, Hb increased to 11.0 g/dL, and LDH normalized in 2–3 weeks after eculizumab. Since he had negative anti-CFH antibodies and *CFHR1-CFHR3* deletions were also reported to be common in healthy patients (6), his genetic findings were not considered disease-causing, his eculizumab therapy was stopped after five doses, and he had no recurrence of aHUS during the 6-year follow-up. His most recent laboratories show normal CBC, creatinine (0.9 mg/dL), and urinalysis.

### Patient 2

**Figure 2:**
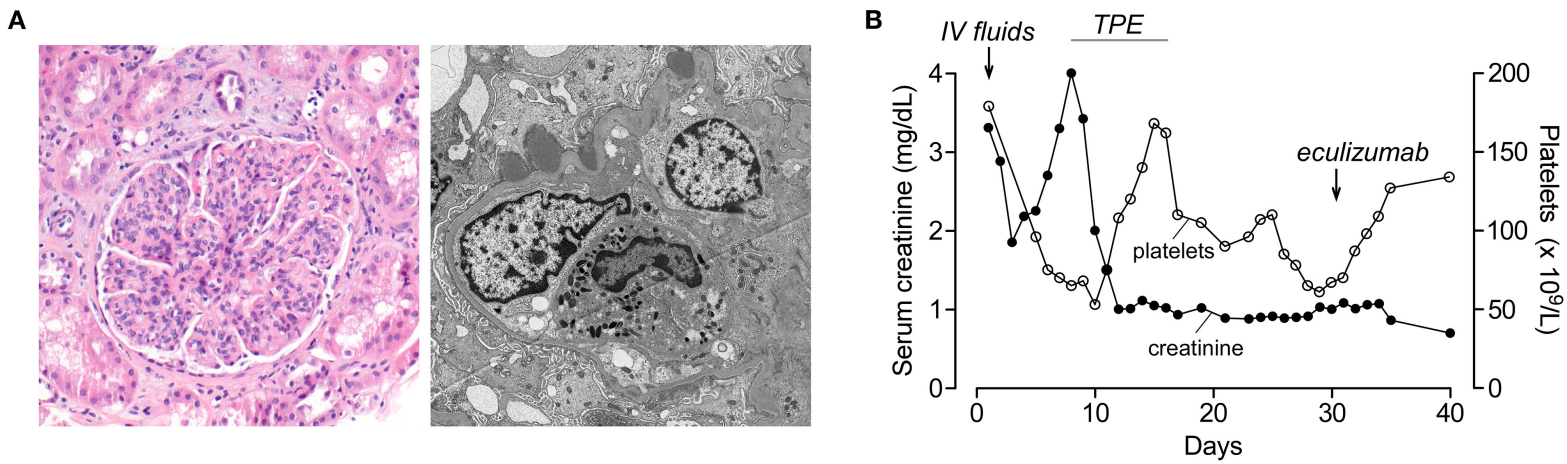
Renal histology and lab results of patient 2 during hospitalization. **(A)** (Left) Hematoxylin and eosin stain (100× magnification) shows a glomerulus with a segmented appearance and prominent endocapillary hypercellularity with frequent neutrophils. Background parenchyma shows occasional injured tubules and scattered interstitial and peritubular neutrophils. (Right) Electron microscopy (4,800× magnification) demonstrates a capillary loop with swollen endothelial cells and endocapillary neutrophils. Several subepithelial hump-like immune complex deposits are present, not associated with significant glomerular basement membrane remodeling. Immunofluorescence microscopy showed IgG (2+), C3 (3+), kappa (2+), and lambda (2+) granular staining along the glomerular capillary loops (not shown). **(B)** Platelet count and serum creatinine during the hospital course. Timing of plasma exchange (TPE) and eculizumab treatments are indicated.

An 18-year-old Asian-American male patient with a history of severe atopic dermatitis presented with intermittent fever for 2 weeks. He appeared ill and dehydrated on examination and had diffuse, erythematous scaly plaques over his upper and lower extremities. Vital signs were significant for hypothermia (35.1°C), normal heart rate, mild tachypnea (20 per min), and hypertension (152/98). His skin culture has recently grown *Group A Streptococcus* and *Staphylococcus aureus*. Due to severe, uncontrolled skin disease along with hypothermia, he was admitted and started on IV antibiotics. Laboratories at admission showed elevated BUN/creatinine (171/3.3 mg/dL), mild anemia (9.9 g/dL), and normal platelets (179 × 10^9^/L). His acute kidney injury (AKI) was initially thought to be due to dehydration, and he was started on IV fluids which improved his creatinine from 3.3 to 1.8 mg/dL on Day 3. His creatinine started to increase on Day 4, and he developed gross hematuria, thrombocytopenia (75 × 10^9^/L), hemoptysis, and respiratory distress on Day 6, requiring ICU transfer. Due to hemoptysis, he underwent bronchoscopy which showed diffuse alveolar hemorrhage. For suspected pulmonary-renal syndrome, he was started on TPE and pulse methylprednisolone on Day 8. His immunological workup was notable for low C3 (8 mg/dl), normal C4, positive anti-streptolysin O, and anti-DNAase B suggesting post-infectious glomerulonephritis (PIGN) diagnosis. His ANA, ANCA, and anti-GBM were negative. His AKI continued to worsen with oliguria, and he was started on intermittent hemodialysis on Day 9. Although he had anemia (7.7 g/dl) and thrombocytopenia (53 × 10^9^/L) at that time, his haptoglobin was normal with no hemolysis findings in the peripheral smear. Due to diagnostic uncertainty, a renal biopsy was performed on Day 12 which showed no signs of TMA but diffuse endocapillary proliferative glomerulonephritis with subepithelial hump-like immune complex deposits consistent with PIGN (Figure 2A). One week after initiation of methylprednisolone and TPE, his creatinine improved to 1.0 and platelets normalized (168 × 10^9^/L). Hemodialysis was stopped, and his serum creatinine continued to improve (nadir 0.7 mg/dL). Consistent with the PIGN diagnosis, his C3 also normalized on Day 25. Starting from Day 27, his creatinine started to increase (peak 1.1 mg/dL) with worsening anemia (hemoglobin 7.4 g/dl), thrombocytopenia (78 × 10^9^/L), and low haptoglobin (<6 mg/dL) with negative Coombs test. Although his peripheral smear did not show schistocytes, in the presence of low haptoglobin, anemia, thrombocytopenia, and acute kidney injury, he was considered to have possible aHUS and started on eculizumab on Day 31 (Figure 2B). One week after eculizumab, his creatinine improved to 0.7 and platelets normalized. His aHUS workup showed normal ADAMTS13 activity, Factor I, Factor H, and Factor B levels, negative anti-CFH antibody and C3 nephritic factor, and negative aHUS genetic testing. He received 10 doses of eculizumab over 15 weeks, and treatment was stopped. He continues to be doing well with no concerns for recurrence of aHUS during the 6-year follow-up. His most recent laboratories show normal CBC, creatinine (0.7 mg/dL), and urinalysis.

## Discussion

Here, we report two severe aHUS cases caused by extremely rare etiologies (Lemierre's syndrome and PIGN). Both patients required kidney replacement therapy and responded to eculizumab with complete renal recovery. Eculizumab was rapidly discontinued in patients with no aHUS recurrence in long-term follow-up.

Infections including *S. pneumonia* and Influenza A are well-known causes of aHUS (1). However, there is only one reported case of aHUS associated with *Fusobacterium necrophorum* bacteremia who had rapid renal recovery in 1 week with antibiotics and TPE (7). Our patient here had worsening renal dysfunction despite antibiotics and TPE, with rapid renal recovery after eculizumab. To the best of our knowledge, this is the first report of eculizumab use in aHUS associated with *Fusobacterium necrophorum* bacteremia and Lemierre's syndrome. Interestingly, our patient had homozygous *CFHR1-CFHR3* deletion which is typically associated with anti-CFH antibodies but also seen commonly in healthy individuals with a population frequency of 33.7% in Sub-Saharan Africa (our patient is an African-American) (5, 6). Considering the negative anti-CFH antibody result, the *CFHR1-CFHR3* deletion in our patient was considered clinically insignificant and he did not have aHUS recurrence during the 6-year follow-up.

Although aHUS and PIGN are both caused by alternative complement pathway dysregulation, they are rarely seen together (8, 9). In the earlier reports, aHUS and PIGN were presented concurrently (10). Our patient here presented with severe PIGN and pulmonary-renal syndrome requiring hemodialysis with no laboratory or histological findings of aHUS. The presentation of our patient with pulmonary-renal syndrome might be considered atypical; however, PIGN is an extremely rare but known cause of pulmonary-renal syndrome (11). Although his PIGN responded to methylprednisolone and TPE with normalization of C3 in <4 weeks, he developed aHUS shortly after that. He had an excellent response to eculizumab which was quickly discontinued with no aHUS recurrence during the 6-year follow-up. To the best of our knowledge, this is the first report of aHUS seen during or after recovery of PIGN. There is one case report for eculizumab use in PIGN-associated aHUS which was discontinued in 1 year with no aHUS recurrence during the 6-month follow-up (9). In our patient here, eculizumab was discontinued at 4 months with no aHUS recurrence during the long-term follow-up. Although the patients did not fit the strict definition of aHUS, they both benefited from eculizumab. Patients and their families were very pleased about being discharged without requiring outpatient dialysis or any obvious long-term sequela. We acknowledge that eculizumab is an expensive treatment; however, its use in selected patients can potentially be a cost-effective approach when compared with the high costs associated with prolonged hospitalization, chronic kidney disease, and renal failure.

An important problem regarding eculizumab use in aHUS is the timing of treatment discontinuation. A recent study suggested that eculizumab can be discontinued in selected aHUS patients with close monitoring; however, recurrence rates were higher in patients with rare variants in complement genes (12). Although they are not classified as disease-causing, complement gene variants could play a role as disease modifiers in cases of aHUS triggered by severe infection or autoimmune glomerulonephritis and potentially impact eculizumab response. In our study, patients did not have rare complement variants, which enabled rapid eculizumab discontinuation with no long-term aHUS recurrence. However, we acknowledge that eculizumab discontinuation in patients with rare complement variants can be challenging due to recurrence risk. We also would like to note that the interpretation of aHUS genetic testing results is not always straightforward due to commonly detected variants of unknown significance (13). Considering all these factors, eculizumab withdrawal should be tailored to the individual patient with different monitorization strategies depending on the risk and resources. For patients requiring long-term complement inhibition, newer agents such as the long-acting anti-C5 monoclonal antibody ravulizumab can be considered (14). In addition, various complement inhibitors including oral drugs are currently in clinical development.

In conclusion, eculizumab can be effective in aHUS associated with rare infectious and autoimmune etiologies and potentially discontinued in the absence of rare complement variants.

## Data availability statement

The original contributions presented in the study are included in the article/supplementary material, further inquiries can be directed to the corresponding author.

## Ethics statement

Ethical review and approval was not required for the study on human participants in accordance with the local legislation and institutional requirements. Written informed consent from the patients was not required to participate in this study in accordance with the national legislation and the institutional requirements. Written informed consent was obtained from the individual(s) for the publication of any potentially identifiable images or data included in this article.

## Author contributions

SS and OC contributed to conception and design of the study and wrote parts of the manuscript. SS wrote the first draft of the manuscript. SS, AU, and OC organized the figures. All authors contributed to manuscript revision, read, and approved the submitted version.
